# A new prescription model for regional citrate anticoagulation in therapeutic plasma exchanges

**DOI:** 10.1186/s12882-017-0494-9

**Published:** 2017-03-01

**Authors:** Sébastien Kissling, Cécile Legallais, Menno Pruijm, Daniel Teta, Bruno Vogt, Michel Burnier, Eric Rondeau, Christophe Ridel

**Affiliations:** 1 Service d’Urgences néphrologiques et Transplantation rénale (UNTR), Hôpital Tenon, Paris, 75020 France; 2 Biomécanique et Bioingénierie, Université de Technologie de Compiègne (UTC), UMR CNRS 7338, Compiègne, 60203 France; 3 Service de Néphrologie et Hypertension, Centre Hospitalier Universitaire Vaudois (CHUV), Rte du Bugnon 17, 1011 Lausanne, CH Switzerland

**Keywords:** Citrate, Clinical protocol, Drug prescription, Mathematical model, Plasma exchanges

## Abstract

**Background:**

Regional citrate anticoagulation (RCA) is proposed for various extracorporeal purification techniques to overcome the risk of bleeding that might result from systemic anticoagulation. Yet, no individualized treatment protocol has been proposed for therapeutic plasma exchange (TPE) so far. The objective of this study was to assess the determinants of blood citrate concentration needed and to develop an individualized RCA protocol useful for clinical practice.

**Methods:**

The study population included 14 patients who underwent a total of 47 TPE sessions. Citrate was infused pre-plasmafilter. Post-plasmafilter and systemic plasma ionized calcium concentrations were measured at standardized time intervals. An algorithm was proposed for the supplementation of calcium. During the discovery phase, citrate was infused at a fixed starting rate, and adapted accordingly to obtained post-plasmafilter ionized calcium levels. Using a mathematical approach, an algorithm was thereafter developed for individualized prescriptions of citrate.

**Results:**

Pre-treatment values of hematocrit and plasma ionized calcium were the main determinants of the required rate of citrate infusion. These can be integrated into a final equation enabling to individualize the prescription. A prefilter ionized calcium concentration between 0.24 and 0.33 mmol/l prevented coagulation of the extracorporeal circuit. Significant hypocalcemia occurred in 8.5% of treatments. There were no significant acid–base disturbances.

**Conclusion:**

We propose a new protocol, which enables for the first time to individualize the prescription of regional citrate anticoagulation during TPE, in an efficient manner. The immediately obtained regional anticoagulation protects against both the risk of coagulation of the membrane and the exposure to an excess of citrate.

**Electronic supplementary material:**

The online version of this article (doi:10.1186/s12882-017-0494-9) contains supplementary material, which is available to authorized users.

## Background

Therapeutic plasma exchange (TPE) is a method of extracorporeal treatment that allows the subtraction and the replacement of the plasma fraction of blood. Thus, TPE represents a powerful tool for the rapid clearance of various pathogenic factors, before drugs exert their beneficial effects [1].

Anticoagulation of blood in the extracorporeal circuit (ECC) is of major importance during TPE, due to close blood-biomaterial interaction. Systemic heparin administration is the most frequently used method but it is contra-indicated in case of high bleeding risk. Regional anticoagulation -hence anticoagulation of the ECC but not of the patient- is a valuable alternative in these situations [2]. Regional anticoagulation with citrate (RCA) is achieved by pre-filter infusion of citrate in the proximal ECC. Citrate binds to ionized calcium in plasma, induces a local hypocalcaemia, which prevents the activation of the coagulation cascade. As a result of post-filter calcium administration and dilution, coagulation properties are restored when the blood returns to the patient. Given the role of ionized calcium in the activation pathways of thrombocytes, polymorphonuclear cells and the complement, biocompatibility may also improve during RCA [3].

Scientific reports on the use of RCA in filtration TPE are scarce [4], despite its rather widespread use in clinical practice [5]. RCA is difficult to apply during TPE for several reasons. Firstly, a given infusion of citrate may lead to a large variability in the plasma ionized calcium concentration of the ECC (cCai). This often requires serial cCai measurements in order to modify the intensity of RCA. Secondly, the balance between the risk of ECC clotting following insufficient citrate infusion and the risk of citrate-related adverse events (systemic hypocalcemia, metabolic acidosis, or alkalosis) may be difficult to reach. This narrow therapeutic window is magnified in TPE because of the convective nature of this method. Indeed, no more than 30% of pre-filter citrate is cleared through the plasmafilter, whereas in comparison about 85% is removed by diffusion during hemodialysis with RCA [6, 7]. Thirdly, caution is warranted during TPE in case of the infusion of fresh frozen plasma (FFP) as replacement solution because FFP contains 17–21 mmol of citrate per liter solution [8]. Most treatment protocols so far do not follow an individualized RCA prescription. Treatment algorithms that take into account the biological determinants of the response to citrate in whole blood are therefore needed in order to increase the security profile of RCA for TPE. In this study we assessed the biological determinants of the response to citrate in whole blood and developed an individualized RCA protocol based on mathematical models.

## Methods

### Patients

Regional citrate anticoagulation for TPE was the method of reference for patients with high bleeding risk in the Service of UNTR, Tenon hospital (APHP, Paris). Ethical approval was not necessary for the analysis of the available data given that this was part of a quality improvement and implementation approach in Tenon hospital. Yet, patients consented orally to the plasmapheresis and were informed of its risks and benefits and of the repeated measurements to be performed for safety reasons during the procedure. No supplemental biological sampling was done for the purpose of the study. Data were analysed anonymously.

### Materials and definitions

**Fig. 1:**
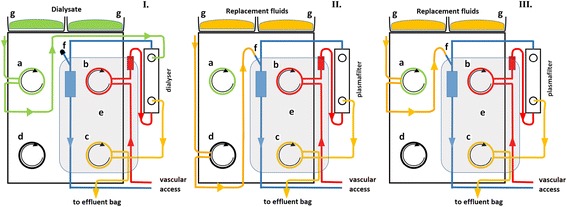
Particularity of extracorporeal circulation (ECC) during TPE using the Ci-Ca module. I. ECC during standard regional citrate anticoagulation (RCA) for continuous hemodialysis treatment. II. ECC during standard therapeutic plasma exchange (TPE). III. ECC during TPE with RCA using the Ci-Ca module and the dialysate pump for plasma replacement. **a** Dialysate pump; **b** Blood pump; **c** Effluent pump; **d** replacement solution pump; e. Kit for continuous hemodialysis treatment (I. and III.), and for TPE treatment (II.); f. Connection to venous bubble trap; g. scale for dialysate solutions (I.) and for plasma replacement solutions

In all patients, TPEs were performed with the Multifiltrate® monitor (Fresenius Medical Care, Germany). The function mode “continuous veno-venous hemodialysis” was selected to take advantage of the module “*Ci-Ca*”. This module offers a dual system of enslavement for the administration of citrate and calcium [4]. The pump dedicated to the administration of citrate is subservient to the blood pump while the effluent pump controls the one dedicated to the administration of calcium. Thus, the regional blood concentration of citrate (and thus the intensity of the RCA) remains constant regardless of the selected blood flow. Also, the administration of calcium is consistently correlated to convective losses of calcium. Additional calcium is administered through an independent calcium pump device as needed. As another particularity, the “*dialysate*” pump was used to administer the substitution solutions (Fig. 1).

In the monitor, the flow rate of the *effluent pump* is dedicated to equalize the sum of each of the following pumps flow rates: the calcium pump, the citrate pump and the substitution pump flow rates. Consequently, this system enables a neutral volume balance. Indeed, there is compensatory excess in plasma filtration related to the administration of the citrate and calcium solutions. This was taken into account for the prescription of the exchange volume and for the choice of the replacement solutions.

**Fig. 2:**
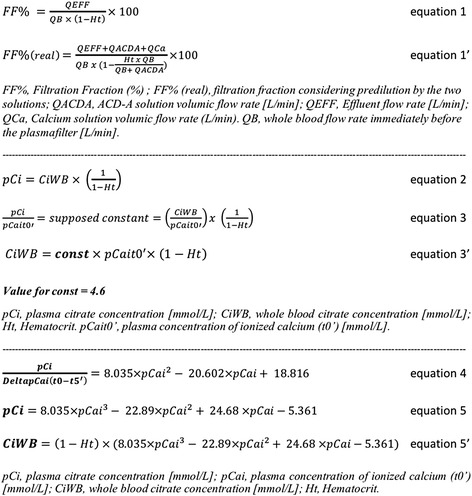
Equations for the progression of the modeling

All the pumps’ flow rates were defined prior to initiation of treatment and were adjusted during treatment as needed. Blood flow rate was fixed at 180–200 mL/min. The maximal flow rate of the substitution pump was 40 mL/min corresponding to a filtration fraction on plasma of 25 to 32% depending on the hematocrit (Ht) and the predilution flow rate (Fig. 2, equation 1 and 1’). The plasmafilter was a PF PSu 2S (polysulfone, surface area 0.6 m^2^, Fresenius medical Care). Gelatin (plasmion®), albumin 5% and FFP were used as replacement solutions.

ACD-A (anticoagulant dextrose-A, citrate 113 mmol/L) was used as regional anticoagulant. ACD-A solution was preferred to a citrate 4% solution because the citric acid component gives 3 hydrogen ions for each citrate molecule and therefore limits the risk of developing significant metabolic alkalosis. Calcium was given through a mixed calcium and magnesium chloride solution. Magnesium chloride was added to compensate for the empirical loss of magnesium induced by multiple successive treatments. In the solution, the molar ratio of magnesium to calcium was 0.36.

Ionized plasma calcium was measured by an ionometer (EH-F, Electrolyte Analyser, Fresenius Medical Care, Germany). Hematocrit and bicarbonate were measured by the central laboratory of the hospital. Clinically significant hypocalcemia was defined as a plasma ionized calcium concentration (pCai) < 0.85 mmol/L with related symptoms or signs at any time point during treatment.

Treatment failure (TF) was defined as the inability to complete a treatment due to blood coagulation in the ECC or as the need to restitute blood to patient because of an ECC clotting threat as announced by a progressive elevation of TMP (trans-membrane pressure). We defined partial treatment failure (PTF) as the elevation of TMP of more than 50 mmHg, which has been correlated to a major risk of ECC clotting in our experience with this membrane. In PTF however, the treatment can be completed.

### Study design

This study was performed in four phases:

- Phase I: the goal was to determine the target range of values for ECC ionized calcium at five minutes treatment (cCai t5’), avoiding TF. Given the short duration of TPE, we initially tested a low citrate concentration aiming a relatively high cCai t5’ of 0.35-0.40 mmol/L. This approach was chosen to minimize the patient exposition to citrate given the convective nature of TPE. Treatment efficacy was characterized by the absence of TF or PTF. The target range for cCai t5’ (corresponding to RCA intensity) should enable treatment efficacy in more than 80% of treatments. In order to assess the determinants of the response to citrate, detailed information were collected.
- Phase II: we tested an empirical approach of the citrate concentration which is required in the ECC to reach the target range of values for cCait5’, and proposed a preliminary protocol that would allow to obtain a maximal number of treatments with cCai t5’ in the target therapeutic range.
- Phase III: Based on data collected during the first and second phases, a mathematical modeling approach has been used to develop the final treatment protocol.
- Phase IV: we assessed clinically the efficacy and safety of the new treatment model.
- Treatment monitoring included serial measurements of characteristic biological parameters at key time points (5, 30, 60, 90 min, end of treatment). This timing was based on the pharmacokinetics of citrate as reported in the medical literature [9]. Additional measurements were performed if deemed necessary by the clinician. Changes in citrate infusion rate were correlated to observed changes in cCai and other biological variables in order to assess the response to citrate. Treatment efficacy was again characterized by the absence of TF or PTF.

### Statistical analysis

Statistical analysis was performed using excel software (Microsoft, Redmond, WA, USA). Quantitative values were expressed as mean ± standard deviation or median values (range), as appropriate. Qualitative variables were expressed as number (percentage).

Polynomial regression analysis was used to predict citrate-induced changes in cCai.

## Results

### Patients

This work included 47 TPE sessions in 14 high bleeding risk patients; each patient received from 1 to 7 treatments. Indications for TPE are shown in Table 1. RCA was indicated because of active bleeding in eight treatments (8/47, 17%). Seven treatments were performed in three patients after renal transplantation.

**Table 1:** Demographic and clinical characteristics of the study patients and treatments

Characteristics of the patients	
Number of patients	14
Sex, n, Female/Male	5/9
Age, years (mean ± standard deviation)	52.6 ± 17.83
Treatment characteristics	
Median number of treatments per patient	2.5
TPE indication (N = 47), n (%)	
Thrombotic microangiopathy	25 (53)
Acute humoral rejection	8 (17)
ANCA-associated vasculitis	7 (15)
Antiglomerular basal membrane vasculitis	3 (6)
Cryoglobulinemia associated vasculitis	2 (4)
Neurological diseases	2 (4)
TPE during active bleeding, n (%)	8 (17)

### Treatment characteristics

Blood flow was 200 mL/min, corresponding to a mean plasma flow of 153 mL/min. Substitution rate was 2.4 L/h. Mean total treatment time was 106.9 (*±*25.1 SD) minutes. Mean exchange volume per treatment was 4.20 (*±*0.72 SD) liters corresponding to 62.4 mL/kg/body weight. The high prevalence of anemia accounts for this relatively high volume exchange. Haven taken into account the predilution volume (which is compensated by plasmafiltration), mean total exchange volume was 4.96 (*±*0.88 SD) liters. However, this supplemental exchange volume is somewhat mitigated by the dilution process. Most treatments (35/38, 92%) included FFP, in 11/14 patients. This was due the high prevalence of thrombotic microangiopathy in the study population and because most of the patients were either bleeding or at high risk of bleeding. FFP constituted more than 80% of total substitution volume during TPE for thrombotic microangiopathy. In other conditions, FFP constituted between 20% (high bleeding risk) and 60% (ongoing bleeding) of total prescribed substitution volume. In 38 TPE, 17 (44.7%) included albumin 5% and 14 (36.8%) included both FFP and albumin 5%. A gelatin solution (Plasmion®) constituted nearly all the non-albumin and non-FFP fraction. Use of sodium chloride was marginal. Altogether, albumin-rich replacement solutions (albumin and FFP) constituted 83.2% of total prescribed substitution volume. When considering the added predilution solutions (ie ACD-A, calcium), the albumin-rich replacement solutions constituted 72.8% of total exchanged volume.

- Phase I: determination of the target range values for cCai t5’, avoiding TF.
  A standardized prescription of citrate and calcium was tested in the first five TPEs performed in three patients, Citrate was perfused pre-filter at a concentration of 2.4 – 3.0 mmol/L of whole blood, aiming a target cCai of 0.35-0.40 mmol/L which correspond to a relatively low concentration as mentioned earlier.
  This infusion rate led to the predefined cCai target, but was associated with TMP elevation and TF (Table 2). For the next treatments, we therefore lowered the cCai target range to 0.25-0.35 mmol/L, which corresponds to the target mostly reported in the literature in various modalities of RRT [6, 10, 11]. This range, deemed appropriate, has been tested in the next successive treatments.

**Table 2:** First 5 TPE aiming a cCait5’ of 0.35-0.40 mmol/L

N° Pt. Ttt	Ht (%)	pCai t0’	CiWB	cCai t5’	cCai max	PTF	TF
A.1	40	1.14	2.4	0.40	0.42	Y	N
B.1	27	1.00	2.4	0.37	0.37	Y	Y
B.2	25	1.07	2.4	0.41	0.41	Y	N
B.3	26	0.83	2.4	0.40	0.47	Y	Y
C.1	27	1.27	3.0	0.39	0.39	y	Y

**Fig. 3:**
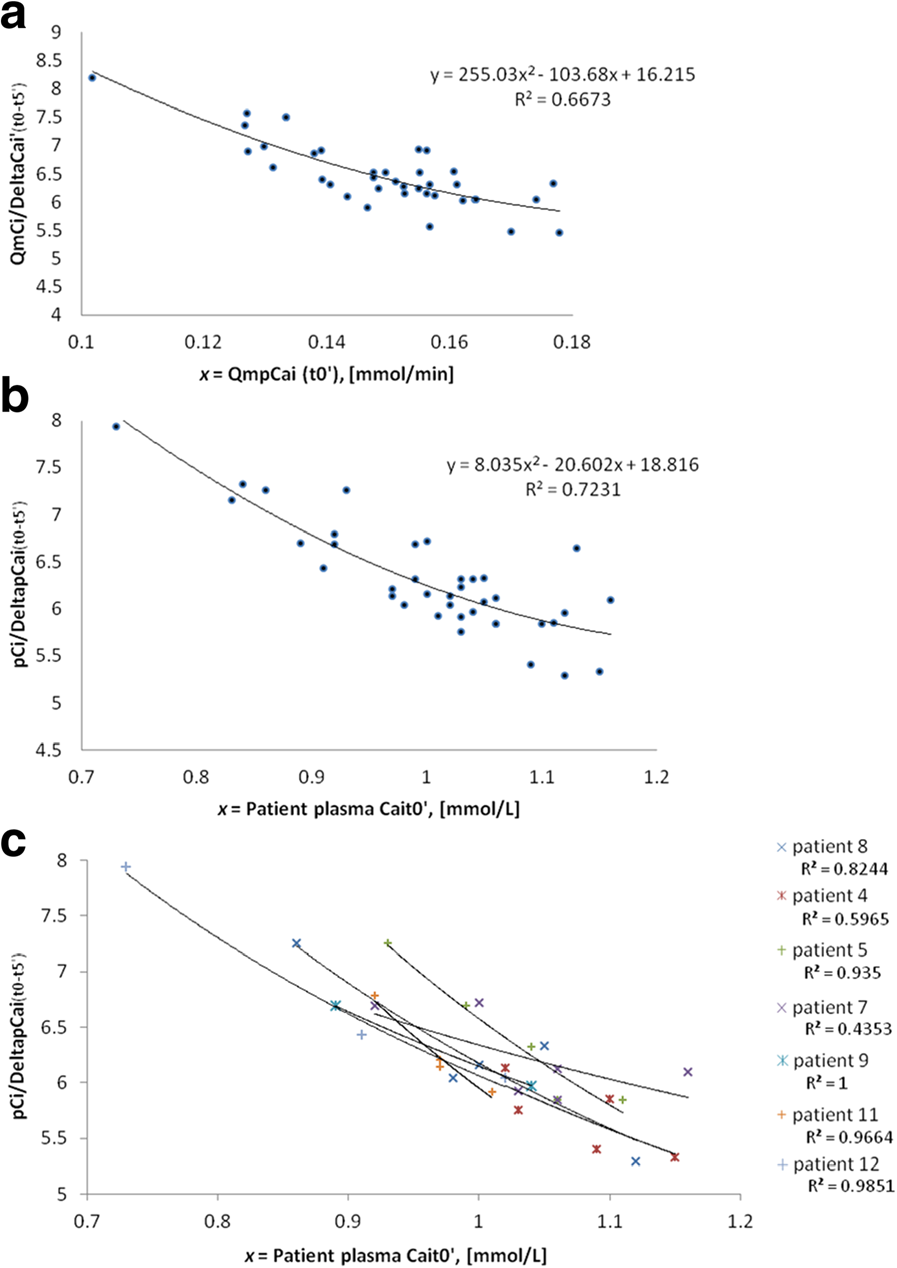
**a** *QmCi/DeltaCai’ as a function of QmpCait0’*. Relation (pre-plasmafilter, t5’) between the ratio « QmCi/DeltaCai’ » (patient massic flow rate of citrate correlated to the induced variation of the massic flow rate of plasma ionized calcium) and « QmpCait0’ » (t0’ massic flow rate of ionized plasma calcium, based on pre-treatment plasma concentration of ionized calcium). **b** ***p*** *Ci/DeltaCai as a function of pre-treatment pCai (pCait0’).* Relation (pre-plasmafilter, t5’) between the ratio « pCi/DeltaCai » (patient plasma ionized calcium concentration correlated to the induced variation (t0’-t5’) of plasma ionized calcium concentration) and pCait0’ (pre-treatment patient plasma ionized calcium concentration). **c** *pCi/DeltaCai as a function of pre-treatment pCai (pCait0’ [mmol/L]).* Same relation as in **b**) per patient in 7 patients
  Using a polynomial regression model, we showed a highly significant correlation between the ratio *pCi*/deltaCai(t0’-5’) and *pCait0’*, in a given individual and for all the treatments taken together (correlation coefficient R^2^ = 0,723, Fig. 3b and c). A similar correlation was noted between massic flow rates of both citrate and calcium (Fig. 3a). This correlation allowed to progress in the development of a prescription protocol (equation 4, Fig. 2).
  In order to obtain an expression of the citrate concentration *pCi* (corresponding to the prescription of citrate in plasma) from *pCait0’*, we introduced empirically in equation 4 (Fig. 2) a value for *cCai* t5’ in the middle of the target range, ie 0.285 mmol/L (equation 5, Fig. 2, Fig. 4).

N° pt.Ttt, Patient.Treatment Number; Ht (%), Hematocrit; pCai, patient ionized plasma calcium [mmol/L]; cCai, circuit plasma ionized calcium [mmol/L]; t0’,t5’, 0, 5 min treatment, respectively; max, maximal value during treatment; CiWB, whole blood citrate concentration [mmol/L]; pCi, plasma citrate concentration [mmol/L]; PTF, partial treatment failure; TF, treatment failure
- Phase II: Empirical approach of the citrate concentration needed in the ECC
  For the next treatments, we empirically increased WBCi to 3.6 mmol/L in order to reach the new target range for cCai t5’ (ie 0.25-0.35 mmol/L). Based on physiological considerations and an analysis of the first treatments parameters, pre-treatment pCai and the Ht were strongly associated with the efficacy of a given dose of citrate. Indeed, since citrate is prescribed in whole blood according to the blood pump and does not enter the erythrocyte, its plasma concentration is assumed to depend on the patient's Ht (Fig. 2, equation 2).
  Concerning pCai, we observed that the citrate concentration required in plasma to lower ionized calcium concentration by one millimole per liter (equivalent to the ratio pCi/pCait0’), was about 4.6 millimoles. Based on the assumption that this value is constant, we developed a first prescription protocol for RCA, which integrated cCait0’ and Ht (Fig. 2, equation 3 and 3’).
  The objective of this preliminary protocol was to obtain a large number of treatments in the target therapeutic range for cCai t5’, that would allow later the development of a mathematical modeling.
  Thus, this preliminary protocol or, alternatively a fixed citrate dosing (WBCi) of 3.6 mmol/L was applied in the next 42 following treatments beyond the first step. Among these, 38 were sufficiently well documented for the plasma values of both pre-treatment ionized calcium and circuit ionized calcium. These 38 TPE could be therefore included in the next phase (modeling process).
  In these 38 TPE, no TF was observed. Aside from two cases (2/38, 5.3%) of PTF that occurred in relation with technical problems (catheter dysfunction, interruption of RCA), any rise of the TMP (4/38, 10.5%) was systematically associated with at least one measurement of cCai above or equal to 0.35 mmol/L at any time during the treatment. Therefore, we adjusted the target range of values for cCait5’ from 0.25-0.35 to 0.24-0.33 mmol/L.
- Phase III: Modeling approach
  When circuit plasma ionized calcium concentration is within the target range (0.24-0.33 mmol/L, in 30 out of 38 treatments), the ratio *pCi/pCait0’* expresses the amount of citrate (in mmol) in one liter of plasma that is required to neutralize one millimole of calcium. As mentioned previously, we assumed that *pCi/pCait0’* was constant (4.6 mmol/mmol) and thus may be used for the calculation of whole blood citrate dosage in any individual patient, knowing pre-treatment *pCait0’* and *Ht* (Fig. 2, equation 3’).
  We further analysed *pCi/pCait0’* and the ratio *pCi/DeltaCai*, where *DeltaCai* is the difference between *pCait0’* and *cCai* t5’. *DeltaCai* also represents the amount of calcium becoming chelated by citrate in 1 l of plasma.
  For relatively higher pre-treatment *pCai*, we found that the ratio *pCi/DeltaCai* is lower (Fig. 3a, b, c). Indeed, less citrate in regard to calcium is needed to reach the therapeutic target. This may be explained by a larger availability of plasma calcium for citrate in case of high plasma calcium concentration. Accordingly, pre-treatment hypocalcemia was associated with a relative resistance to citrate treatment.

**Fig. 4:**
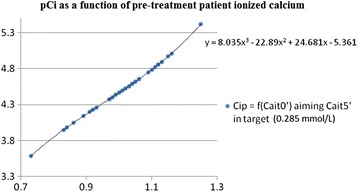
Theoretical plasma citrate concentration (pCi) that is needed to obtain therapeutic postfilter anticoagulation (c*Ca*_*i*_ *t5’, 0.285 mmol/L*) and its relation to patient pre-treatment plasma ionized calcium concentration (Cait0’). Third degree polynomial equation derived from the polynomial second degree expression of the ratio *Cip/DeltaCai*
  Equation 5’, illustrated in Fig. 2, integrates the *Ht* and may be used for the determination of the initial dose of citrate in whole blood.
- Phase IV: Clinical safety and efficacy analysis
  The retrospective application of this new formula to the values of pre-treatment *pCai* of the 47 treatments of the study, allowed to improve both citrate underdosing (*cCai* at t5' > 0.33 mmol/L in 4 treatments) and overdosing (*cCai* at t5' <0.24 mmol/L in 4 treatments). This was at the price of a slight rise in citrate dosing in 6 treatments during which dosing was already adequate.
  Concerning safety, we managed to adapt calcium infusion according to a pre-defined protocol. A cautious clinical monitoring and the timing of the serial measurements of *pCai* allowed for the identification of biological anomalies which were corrected with the application of a systematic algorithm (Additional file 1: Table S1 and Additional file 2: Table S2). Clinically significant hypocalcemia was observed in four (8.5%) treatments and were corrected with calcium chloride administration without complication. Total calcium rose significantly during treatment as a consequence of calcium administration to maintain pCai balance (Fig. 5). This was associated with a concurrent elevation of the ratio pCa total/pCai (Table 3). As expected, the highest values of the pCa total/pCai ratio were observed during TPE with large amount of FFP as the substitution solution. Unexpectedly however, there were no proportional differences in the amount of the calcium infused nor in the concentration of post-treatment pCai (Fig. 6).

**Fig. 5:**
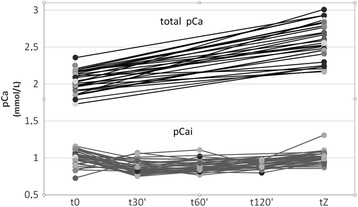
Plasma concentrations of ionized (pCai) and total (total pCa) during treatment. Time 0,30,60,120 min and Z (end) of treatment

**Table 3:** Pre-treatment and post-treatment plasma values of pCai, pCa total, the ratio pCa total/pCai, pH and bicarbonates (mean ± SD))

	Pre-treatment (t0’)	Post-treatment
pCai (mmol/l)	1.00 ± 0.1	1.02 ± 0.07
pCa total (mmol/l)	2.04 ± 0.14	2.58 ± 0.25
pCa total/pCai	-	2.53 ± 0.28
pH	7.47 ± 0.05	7.48 ± 0.04
Bicarbonate (mmol/l)	26.4 ± 4.9	27.6 ± 5.5

pCai, patient ionized plasma calcium; pCa total, patient plasma total calcium

**Fig. 6:**
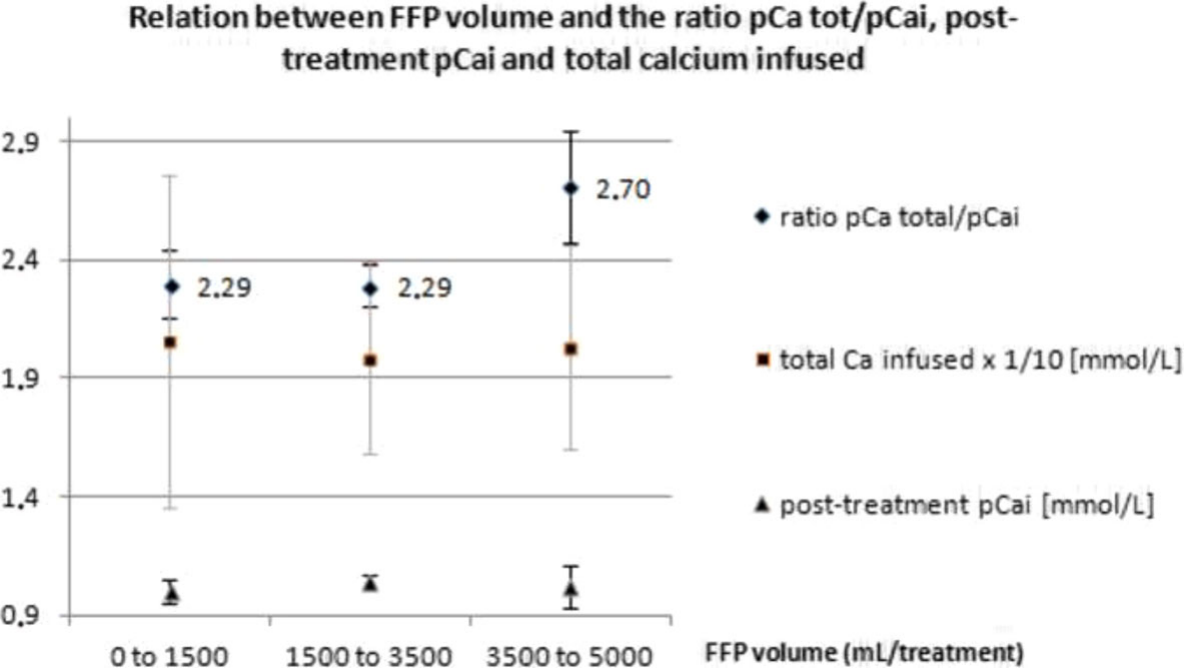
Relation between Fresh Frozen Plasma (FFP) volume and the ratio pCa total/pCai, post-treatment pCai and total amount of calcium infused during RCA TPE
  We did not observe any new episode of bleeding, worsening of a pre-existing bleeding, nor clinically significant electrolytes and acid–base disturbances (Additional file 3). We considered as a clinically significant acid–base disturbance any metabolic alcalosis or acidosis that was complicated by an adverse clinical event requiring a medical intervention. Mean bicarbonate elevation per treatment appeared to be proportional to FFP volume, as was pH elevation (Fig. 7). In four patients undergoing 5 to 6 daily consecutive TPE using FFP for more than 80% of total replacement solutions (mean total FFP = 21 L *±* 3.71 SD), mean bicarbonate variation throughout the period of treatment was significant (+14.5 mmol/L *±* 8.76 SD, from an initial mean bicarbonate concentration of 20 *±* 4.32 SD mmol/L). However, mean pH variation was relatively modest (+0.1 *±* SD 0.06 units). None of them required medical intervention.

**Fig. 7:**
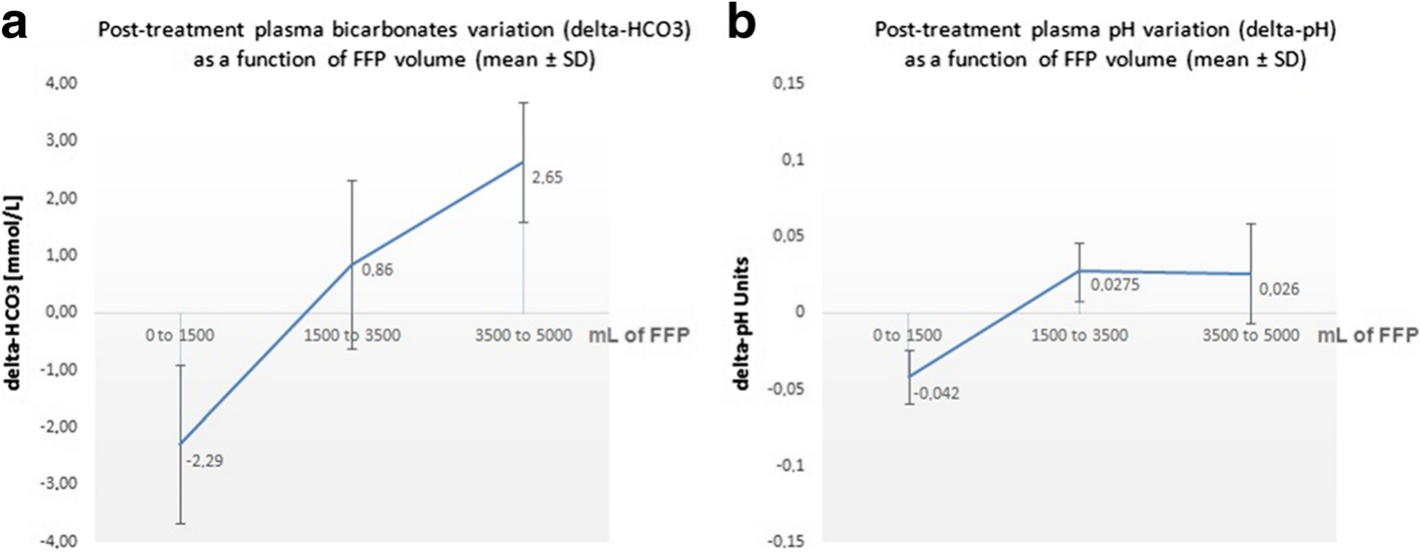
Mean values (and standard deviation, SD) for treatment-induced variation in (**a**) plasma bicarbonate concentration (delta-HCO_3_), and (**b**) plasma pH (delta-pH). From 38 therapeutic plasma exchanges. Delta-HCO_3_ appeared proportional to the volume of FFP infused as a replacement solution

## Discussion

Taken together, this report describes a new, individualized prescription protocol for RCA based on the pre-treatment *pCai* and *Ht*. The pivot of the work is to demonstrate how to develop a mathematical model built to improve and individualize the prescription of citrate infusion, namely the concentration of citrate in whole blood that is actually required to optimize the treatment.

We first defined the most adequate concentration of calcium in the circuit which should be between 0.24 and 0.33 mmol/l. Our data show that there is a close correlation between the needed citrate concentration in whole blood and pre-treatment values of *pCai* and hematocrit. Thus, the application of this model should enable to propose a more individualized prescription of RCA in TPE.

Regional citrate anticoagulation is particularly difficult to apply in patients undergoing TPE because of the narrow therapeutic window and the risk of citrate overdosing. This is probably the main reason why this method is not commonly used in clinical practice. Indeed, there is no literature on the habits relative to the use of RCA for TPE in apheresis centers worldwide. Another reason for its limited use might be the labor intensity associated with RCA.

As mentioned previously, we have evaluated whether a relatively high calcium concentration in the ECC would allow treatment efficacy. Our experiments stress that cCai values should be lower than 0.33 mmol/L to insure efficacy. This is in accordance with the fact that a very close blood-biomaterial interaction precludes any compromise on the intensity of anticoagulation during TPE. Accordingly, achieving immediately effective anticoagulation should protect against the risk of clotting of the system.

Our study strongly also suggests that a fixed universal prescription of citrate in whole blood is not appropriate since it could lead to a higher number of ECC clottings. According to our model, this would be especially the case in anaemic and hypercalcemic patients. In this work, we illustrate that pre-treatment Ht and circuit ionized calcium concentration (cCai) are the strongest predictors of the amount of needed citrate. This is not surprising given the fact that citrate does not enter the erythrocyte and that pCai is chelated by citrate.

Regarding the efficacy, our data suggest that the model should enable a better prediction of the amount of needed citrate, thanks to the individualization of the prescription. Yet, this needs validation in prospective studies.

As such, the application of this protocol should limit both the risk of citrate overdosing and the risk of clotting of the ECC. Furthermore, and of most importance during TPE, the application of this model is likely to reduce the time to reach target *cCai*. So, this protocol has the potential to reduce the labor-intensity of RCA-based TPE.

The patients we treated showed a high incidence of citrate accumulation as defined by a *pCa total/pCai ratio* > 2.5 [12]. Obviously, hypocalcemia that results from citrate accumulation, rather than the citrate plasma concentration by itself, is the causative factor of citrate toxicity. We think therefore that citrate accumulation should not be used as a surrogate for citrate intoxication without looking closely at pCai and plasma bicarbonate concentrations. Furthermore, we should underscore that the pCa total/pCai ratio is an “a posteriori” indicator of citrate accumulation that cannot be used to monitor the treatment in real time during TPE as it is during a slow continuous blood purification method. Moreover during filtration TPE, citrate accumulation is due mostly to a high citrate flow rate to the patient rather that to a defective metabolism. This is further supported by a post-treatment rise in plasma bicarbonate concentration mostly in case of a high amount of substitution by FFP (Fig. 7).

Although we observed a relatively high rate of ionized hypocalcemia in this study, this cannot be attributed to the RCA prescription model by itself. We believe that this was merely due to the high blood flow (ie 200 mL/min), which exposes the patient to a high citrate load. This hypothesis is supported by Fig. 6, which suggests that the dependence of citrate accumulation (i.e. pCa total/pCai > 2.5) on the volume of FFP is not associated with a simultaneous proportional excess in the incidence of post-treatment hypocalcemia nor a larger amount of calcium infusion. The calcium content of donor FFP most probably gives a part of the explanation for this interesting observation.

In the future, a reduction of the blood flow should lessen the citrate load without changing the validity of the protocol. The substitution solution also plays an important role in the incidence of hypocalcemia. When FFP are administered as a substitute, the infusion of the minimal effective dose of citrate is crucial. Indeed, FFP contains large amounts of citrate with no anticoagulant value. Therefore, in order to limit the patient exposition to citrate, the flow of the blood pump should be lowered to 120–150 mL/min during plasma substitution with FFP. At the same time, the rate of replacement should not exceed 1600 mL/h. Given the small number of treatments (only four with PTF), we were not able to demonstrate any difference in the incidence of PTF between treatment with and without FFP. However, PTF seemed not to be associated with the use of FFP (no PTF observed) nor with the duration of treatment (106.25 min for the 4 TPE showing PTF vs 106.9 min).

Whatever the modality of treatment prescription and monitoring, application of RCA for TPE requires a great expertise of both the medical and the nurse team. In particular, excellent knowledge is necessary in the fields of electrolytes disorders and citrate metabolism. Furthermore, everyone who prescribes or manages such treatment must thoroughly know how to handle the functionality of the monitor and how to react in case of alarms.

This study must be seen in the light of its strengths and limitations. Among its strengths are the use of the same experienced treatment team throughout the study, and the use of a discovery and a development phase with a mathematical approach. Among its limitations are the relatively limited number of TPE performed in this work and the heterogeneity of the treatment indications. Besides, only few treatments were available within the highest ranges of calcium levels. Therefore, there might have been some degree of bias when this protocol was applied in the upper fields of pre-treatment pCai values.

Finally, a local validation of the curve expressing the pCi needed in the ECC as a function of pCait0’ is an absolute prerequisite before starting using this prescription protocol. Most ionometers are indeed not validated to measure cCai in the range of values required for RCA. In our experience, we observed that the application of this protocol with some others ionometers could lead to a systematic under dosing of the pCi. This argues for the need to calibrate individually the prescription formula with the local ionometer. However, these limitations should not prevent the application of this protocol at a large scale.

## Conclusions

We propose a new model, which enables for the first time to individualize the prescription of RCA during TPE. Application of this new model should result in an improvement of the quality of the treatment by limiting both the risk of citrate overdosing and the risk of clotting of the ECC. This hypothesis needs to be validated in a prospective study.

## Additional files

Additional file 1: Table S1. Table for the adaptation of calcium infusion depending on pCai t30’ and following controls. Remark: quantities of Calcium infusion are not universal, but will vary from one hospital to another, based on local parameters (ionometer, TPE device, applied blood flow). (TIF 113 kb)
Additional file 2: Table S2. Table for the prescription of the initial rate of calcium infusion (dependence on the effluent flow). (TIF 21 kb)
Additional file 3: Figure S1. Post-treatment variation in plasma magnesium (Mg) concentration (x-axis) according to pre-treatment concentration (y-axis). (TIF 87 kb)

## Abbreviations

ECC: Extracorporeal circuit
Ht: Hematocrit
PTF: Partial treatment failure
RCA: Regional citrate anticoagulation
TF: Treatment failure
TMP: Trnsmembrane pressure
TPE: Therapeutic plasma exchange
WBCi: Whole blood citrate
