# Erratum to: A new prescription model for regional citrate anticoagulation in therapeutic plasma exchanges

**DOI:** 10.1186/s12882-017-0500-2

**Published:** 2017-03-09

**Authors:** Sébastien Kissling, Cécile Legallais, Menno Pruijm, Daniel Teta, Bruno Vogt, Michel Burnier, Eric Rondeau, Christophe Ridel

**Affiliations:** 1 Service d’Urgences néphrologiques et Transplantation rénale (UNTR), Hôpital Tenon, Paris, 75020 France; 2 Biomécanique et Bioingénierie, Université de Technologie de Compiègne (UTC), UMR CNRS 7338, Compiègne, 60203 France; 3 Service de Néphrologie et Hypertension, Centre Hospitalier Universitaire Vaudois (CHUV), Rte du Bugnon 17, 1011 Lausanne, CH Switzerland

## Erratum

In the original publication of this article [1], figure 6 contains the inscription "total Ca infused x 1/10 [mmol/l], this inscription was not correct and should have read "total Ca infused x 1/10 [mmol]. The original figure 6 (Fig. 1) and the corrected figure 6 (Fig. 2) are published in this erratum.

**Fig. 1:**
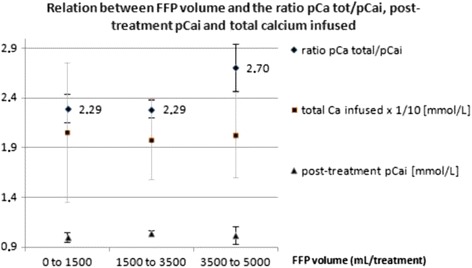
original version of figure 6 as published on 1 March 2017

**Fig. 2:**
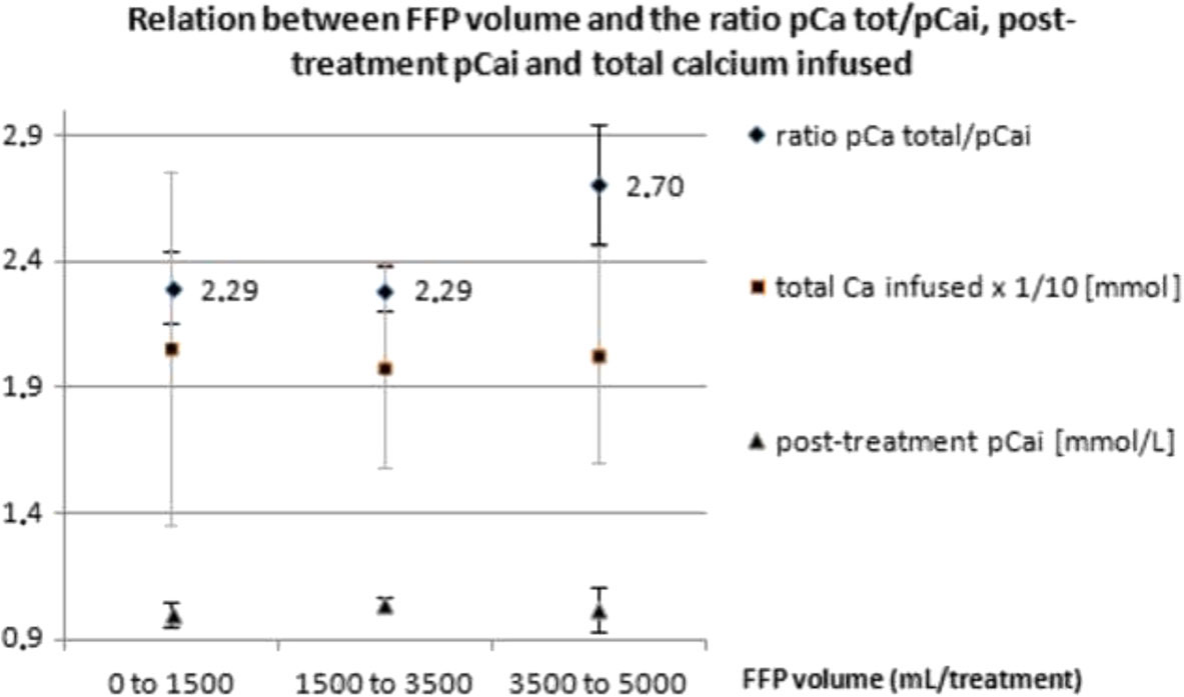
corrected version of figure 6
