# Supplementary figures and images for: A new prescription model for regional citrate anticoagulation in therapeutic plasma exchanges
Source: BMC Nephrol. 2017 Mar 1;18:81. doi: 10.1186/s12882-017-0494-9 (PMC5333425; doi:10.1186/s12882-017-0494-9)

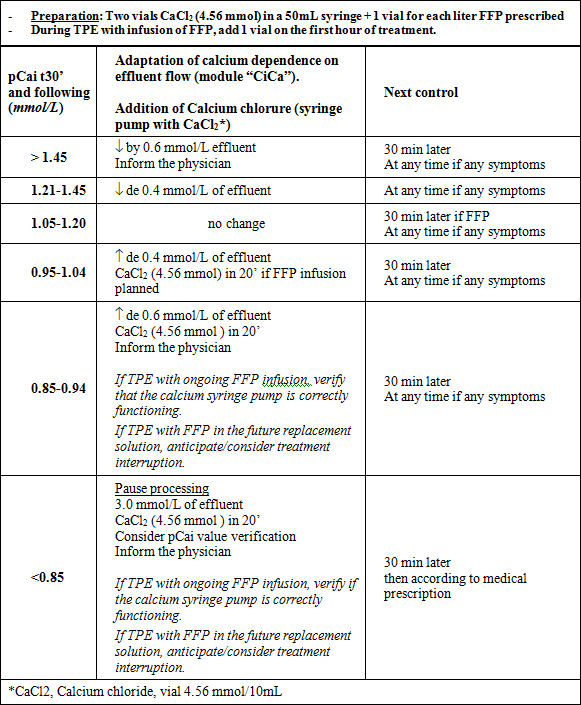

Supplement: Additional file 1: Table S1. — Table for the adaptation of calcium infusion depending on pCai t30’ and following controls. Remark: quantities of Calcium infusion are not universal, but will vary from one hospital to another, based on local parameters (ionometer, TPE device, applied blood flow). (TIF 113 kb) [file 12882_2017_494_MOESM1_ESM.tif]

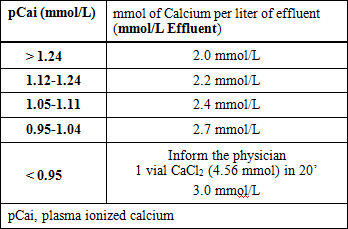

Supplement: Additional file 2: Table S2. — Table for the prescription of the initial rate of calcium infusion (dependence on the effluent flow). (TIF 21 kb) [file 12882_2017_494_MOESM2_ESM.tif]

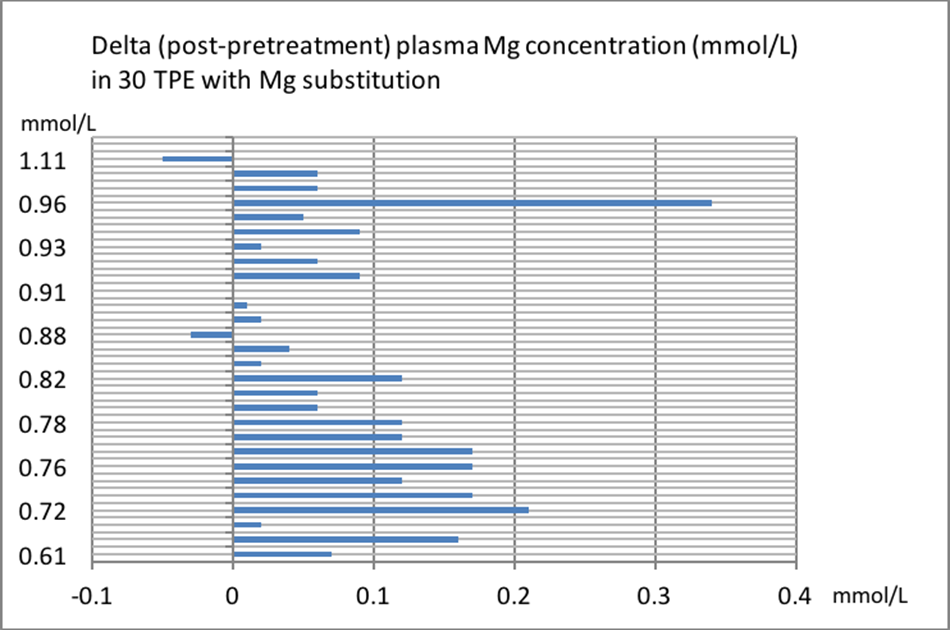

Supplement: Additional file 3: Figure S1. — Post-treatment variation in plasma magnesium (Mg) concentration (x-axis) according to pre-treatment concentration (y-axis). (TIF 87 kb) [file 12882_2017_494_MOESM3_ESM.tif]
